# Contemporary national treatment patterns for clubfoot relapse-related interventions: a large multi-institutional cohort

**DOI:** 10.1186/s12891-026-10032-7

**Published:** 2026-05-28

**Authors:** Christopher D. Hamad, Joshua Wiener, Timothy Liu, Autreen Golzar, Al-Hassan J. Dajani, David C. Kaelber, Nicholas M. Bernthal, William L. Sheppard, Soroush Baghdadi, Mauricio Silva

**Affiliations:** 1 Department of Orthopaedic Surgery, University of California, 1225 15th Street – Suite 3144B Santa Monica, Los Angeles, CA 90404 USA; 2 Case Western Reserve University School of Medicine, Cleveland, OH USA; 3 Population and Quantitative Health Sciences, Case Western Reserve University and the Center for Clinical Informatics Research and Education, The MetroHealth System, Cleveland, OH USA; 4 David Geffen School of Medicine, University of California, Los Angeles, CA USA; 5 Luskin Orthopaedic Institute for Children, Los Angeles, CA USA

**Keywords:** Clubfoot, Ponseti method, Recurrence, Serial casting, Tibialis anterior tendon transfer, Pediatric orthopaedics, Epidemiology

## Abstract

**Background:**

While the Ponseti method is the gold standard for treatment of congenital clubfoot, recurrence after initial correction remains common. Most published data describing management of recurrent deformity are derived from single center series, and national practice patterns are not well defined. The purpose of this study was to characterize contemporary utilization of interventions used as proxies for clubfoot recurrence in a large multi-institutional database.

**Materials and methods:**

We conducted a retrospective descriptive study using the TriNetX Research Network. Children diagnosed with idiopathic clubfoot at one year of age or younger were identified and followed longitudinally for up to seven years after an index ambulatory visit. Relapse related interventions were defined using CPT codes and included repeat casting, repeat Achilles tenotomy, tibialis anterior tendon transfer, and bony osteotomies. Kaplan Meier analyses were used to estimate the cumulative probability of remaining free from each intervention over time.

**Results:**

The final cohort included 4,522 children with early diagnosed idiopathic clubfoot and longitudinal follow up. Any relapse-related intervention occurred in 23.11% of children, with repeat casting being the most common (16.08%). Tibialis anterior tendon transfer was performed in 6.23% and repeat Achilles tenotomy in 5.75% of eligible patients. Bony procedures were uncommon, with calcaneal osteotomy performed in 0.75%, tarsal osteotomy in 1.64%, and first metatarsal osteotomy in 0.27% of children. Kaplan Meier estimates demonstrated that casting and tendon procedures occurred earlier in childhood, whereas osteotomies were rare late interventions with procedural free survival exceeding 97% at seven years.

**Conclusions:**

In this large national cohort of children with idiopathic clubfoot, procedure-based interventions consistent with recurrence occurred in approximately one-quarter of patients, with repeat casting representing the predominant management strategy. Tendon procedures were used selectively, and bony procedures were rare, reinforcing their role as salvage interventions for rigid or multiply recurrent deformities. These population level data provide contemporary benchmarks for counseling families, guiding expectations, and informing future research on recurrence management following Ponseti treatment.

## Introduction

Congenital talipes equinovarus (clubfoot) occurs in approximately 1.18 per 1000 births with nearly 200,000 children affected worldwide [1, 2]. It is a major cause of pain, loss of function, and disability, especially in lower and middle income countries (LMIC) [1].

Ponseti revolutionized clubfoot treatment from a mostly surgical approach with poor to fair outcomes, to a deformity mainly managed by serial casting and minimally-invasive procedures [3–7]. Ponseti method is the gold-standard for treatment of clubfoot, given the scalability, cost-effectiveness, and reproducible results [5]. While extremely successful, clubfoot recurrence is a recognized adverse outcome after initial successful Ponseti casting [6, 8–10]. The reported rates of recurrence vary widely in the literature, from under 5% to over 60% [6, 8–10]. This wide range is partly due to the nature of the condition and loss to follow-up, but also due to the small patient population in the published literature and potential biases in reporting [6, 8–10]. The treatment of clubfoot recurrence has shifted towards a trial of repeat casting, followed by possible individualized surgical intervention, which may include tibialis anterior tendon transfer (TATT), a la carte posterior-medial release, osteotomies, and fusion procedures.

While primary Ponseti treatment has, unsurprisingly, garnered attention and a long publication record, the treatment of recurrences is not discussed as often in the literature. Moreover, the surgeon preferences and their current treatment paradigms for clubfoot recurrence are not fully understood. Much of the available literature describing interventions used as proxies for clubfoot recurrence is derived from single-center case series, which may be limited by small sample size, referral bias, local surgeon preference, institutional treatment philosophy, and differences in brace adherence or follow-up. These factors can produce wide variation in reported intervention rates and limit generalizability. A large multi-institutional database may therefore provide a broader estimate of contemporary national practice patterns and help contextualize findings from individual centers. Therefore, the goal of this study was to analyze a large, national multi-institutional database to determine the current practices in the management of clubfoot recurrence. We hypothesized that repeat casting would be the most common treatment, followed by surgical intervention [5, 11–13].

## Materials and methods

We conducted a retrospective descriptive study using the TriNetX Research Network (Cambridge, MA, USA), a federated platform aggregating de-identified electronic health records from U.S. health systems. The Research Network includes over 160 million patients across 111 healthcare organizations. All data in TriNetX are de-identified under the Health Insurance Portability and Accountability Act (HIPAA) Privacy Rule (45 CFR § 164.514[b] [1]) using expert determination methods and minimum cell sizes (≥ 10). This study does not constitute human subjects research under 45 CFR § 46.102; therefore, institutional review board approval was not required. The TriNetX Research Network was queried on March 26, 2026. This study was conducted in accordance with the Strengthening the Reporting of Observational Studies in Epidemiology (STROBE) guidelines.

We identified children diagnosed with clubfoot using the International Classification of Diseases, Ninth and Tenth Revisions (ICD-9 and ICD-10) codes 754.51 and Q66.0, respectively, at age one year or younger, who subsequently had at least one ambulatory visit between January 1, 2010 and December 31, 2018. Children with syndromic or neuromuscular conditions associated with secondary clubfoot were excluded, including spina bifida (ICD-10 Q05), Down syndrome (Q90), cerebral palsy (G80), muscular dystrophy (G71.0), myotonic disorders (G71.1), congenital myopathies (G71.2), spinal muscular atrophy (G12), and hereditary neuropathy (G60). The date of the qualifying ambulatory visit served as the index event, with eligibility requiring that the clubfoot diagnosis occur at least one year prior to the index visit. The requirement that the index ambulatory visit occur at least one year after the initial clubfoot diagnosis was applied as a temporal safeguard to ensure completion of primary Ponseti treatment, which typically consists of serial casting followed by Achilles tenotomy and post-tenotomy casting within the first 4 to 6 months of life [5]. Additionally, tibialis anterior tendon transfer is rarely performed before 2 years of age, supporting that procedures captured after the index visit are more likely to represent relapse rather than primary treatment. All outcomes were analyzed within a standardized follow-up window beginning 1 day after the index event and continuing for 2,555 days (7 years). The seven-year window represents the maximum follow up period available for Kaplan Meier analysis and was not a mandatory inclusion criterion. This is consistent with relapse being reported between 2 and 8 years of age most commonly. Relapse-related interventions were defined using Current Procedural Terminology (CPT) codes embedded within TriNetX outcome definitions. Outcomes of interest included repeat casting (CPT 29450), repeat Achilles tenotomy (CPT 27605, 27606), tibialis anterior tendon transfer (CPT 27691), calcaneal osteotomy (CPT 28300), tarsal osteotomy (CPT 28304), and first metatarsal osteotomy (CPT 28306). Repeat casting was defined using CPT 29,450, which is specific to clubfoot cast application. The requirement that the index visit occur at least one year after the initial clubfoot diagnosis was intended to reduce capture of primary Ponseti casting and better isolate later casting events. However, CPT coding does not provide the clinical indication for each cast, and therefore casting events may include minor late adjustments or other clubfoot-related management in addition to treatment for clinically documented recurrence. Because clinical recurrence cannot be directly ascertained from administrative data, these procedure-based outcomes were used as proxies for recurrence. The composite outcome “any relapse procedure” was defined at the patient level, with each patient counted once at the time of their first qualifying intervention regardless of the number or type of subsequent procedures. For procedures in which prior exposure could affect interpretation (including TATT and all osteotomies), patients with any record of the procedure before the start of follow up (Day 1 after the index visit) were excluded to ensure only incident relapse procedures were captured. For repeat casting and Achilles tenotomy, no prior procedure exclusion was applied, as these are components of initial Ponseti treatment and exclusion would remove the entire at risk population. Risk analyses estimated the proportion of children undergoing each intervention during follow-up. Kaplan-Meier survival analyses estimated the cumulative probability of remaining free from each intervention over time, censoring patients at their last recorded clinical encounter. All analyses were descriptive, and no propensity matching or comparative testing was performed, as the goal of this study was to characterize relapse management patterns in a large, multi-institutional cohort.

## Results

### Cohort size and demographics

**Fig. 1:**
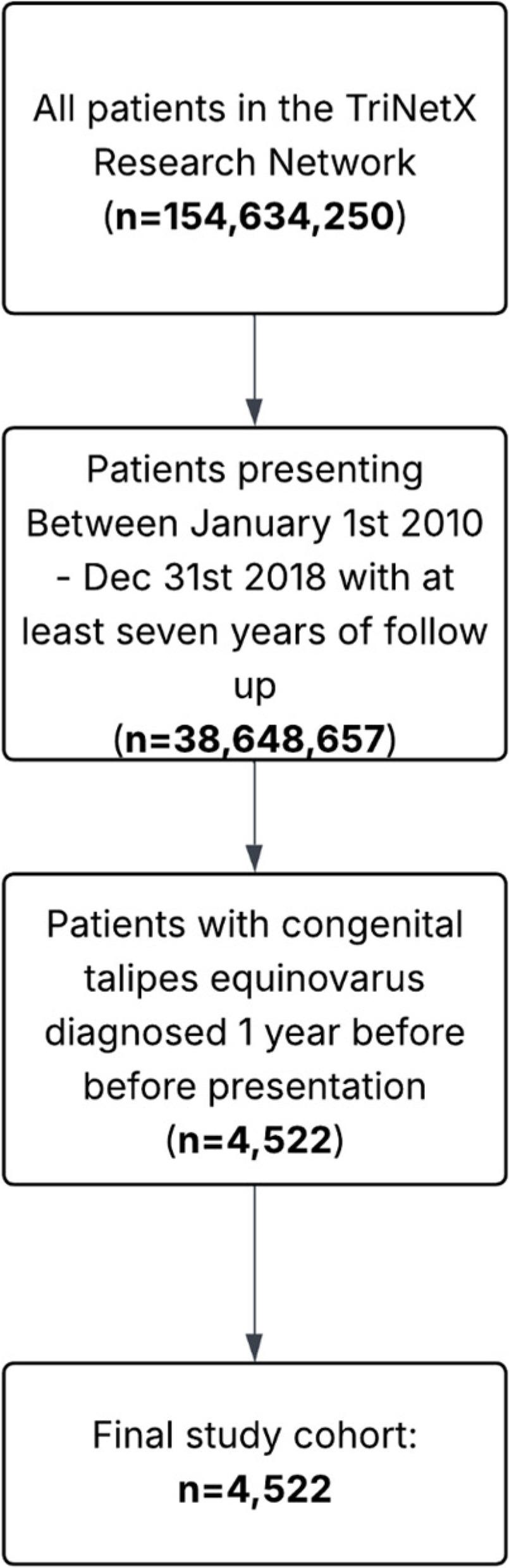
Patient cohort selection diagram. Flow diagram demonstrating identification of children with idiopathic clubfoot within the TriNetX research network. Patients were included if diagnosed at ≤1 year of age and had at least one qualifying ambulatory visit between 2010 and 2018. Exclusion criteria included syndromic and neuromuscular conditions associated with secondary clubfoot. The final analytic cohort consisted of 4,522 children

The final cohort consisted of 4,522 children with early-diagnosed idiopathic clubfoot and up to 7 years of longitudinal follow-up within the TriNetX Research Network (Fig. 1) (Table 1).

**Table 1:** Patient demographics

Characteristic	*n*	%
Total cohort	4,522	—
Age at index visit, years		
Mean ± SD	2.36 ± 3.12	—
Sex		
Male	2,935	65.0
Race		
White	2,946	65.3
Black or African American	510	11.3
Asian	173	3.8
Other	306	6.8
Unknown / Not reported	482	10.7
Ethnicity		
Hispanic or Latino	618	13.7

### Repeat casting

Relapse-related interventions were common, with repeat casting representing the most frequent treatment modality. A total of 727 of 4,522 children (16.08%) underwent repeat casting.

### Achilles tenotomy and TATT

Repeat Achilles tenotomy was also frequently performed, occurring in 260 children (5.75%). TATT occurred in 281 of 4,513 eligible children (6.23%) after excluding 10 patients with prior tendon transfer.

### Osteotomies

Bony procedures for relapse were substantially less common. Calcaneal osteotomy occurred in 34 of 4,514 children (0.75%) after excluding 10 patients with prior calcaneal osteotomy, while tarsal osteotomies occurred in 74 of 4,509 eligible children (1.64%) after excluding 13 patients with prior tarsal osteotomy. First-metatarsal osteotomy was rare, occurring in only 12 of 4,522 children (0.27%).

### Any relapse procedure

**Fig. 2:**
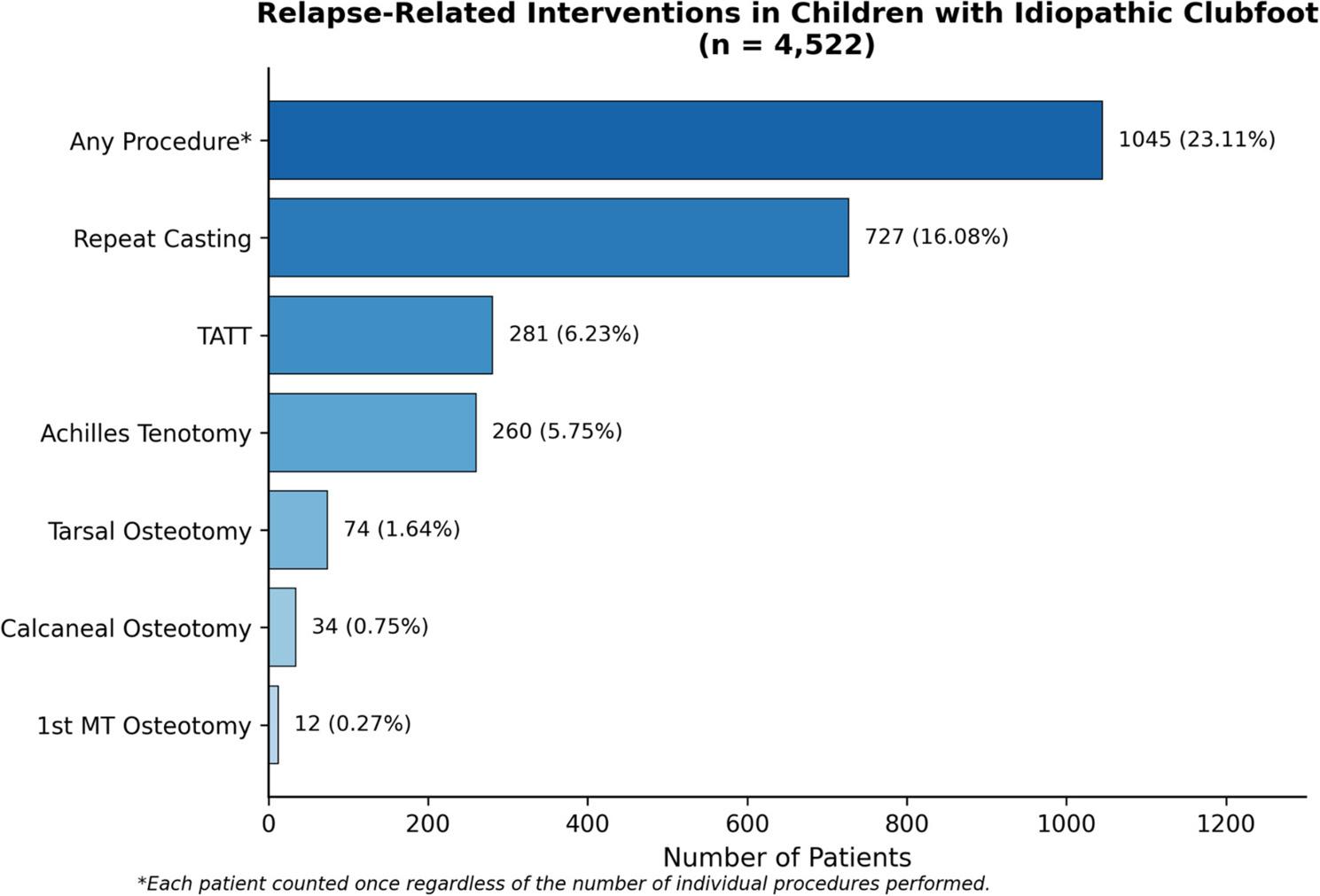
Number of clubfoot relapse interventions. Bar graph illustrating the number of children undergoing each procedure during follow-up. Interventions include repeat casting, repeat Achilles tenotomy, Tibialis Anterior Tendon Transfer (TATT), calcaneal osteotomy, tarsal osteotomy, and first metatarsal osteotomy. Counts represent the number of patients undergoing at least one instance of each procedure during the study period (*N* = 4,522)

Overall, 1,045 of 4,522 children (23.11%) underwent at least one relapse-related procedure during follow-up (Fig. 2).

### Temporality of interventions

**Fig. 3:**
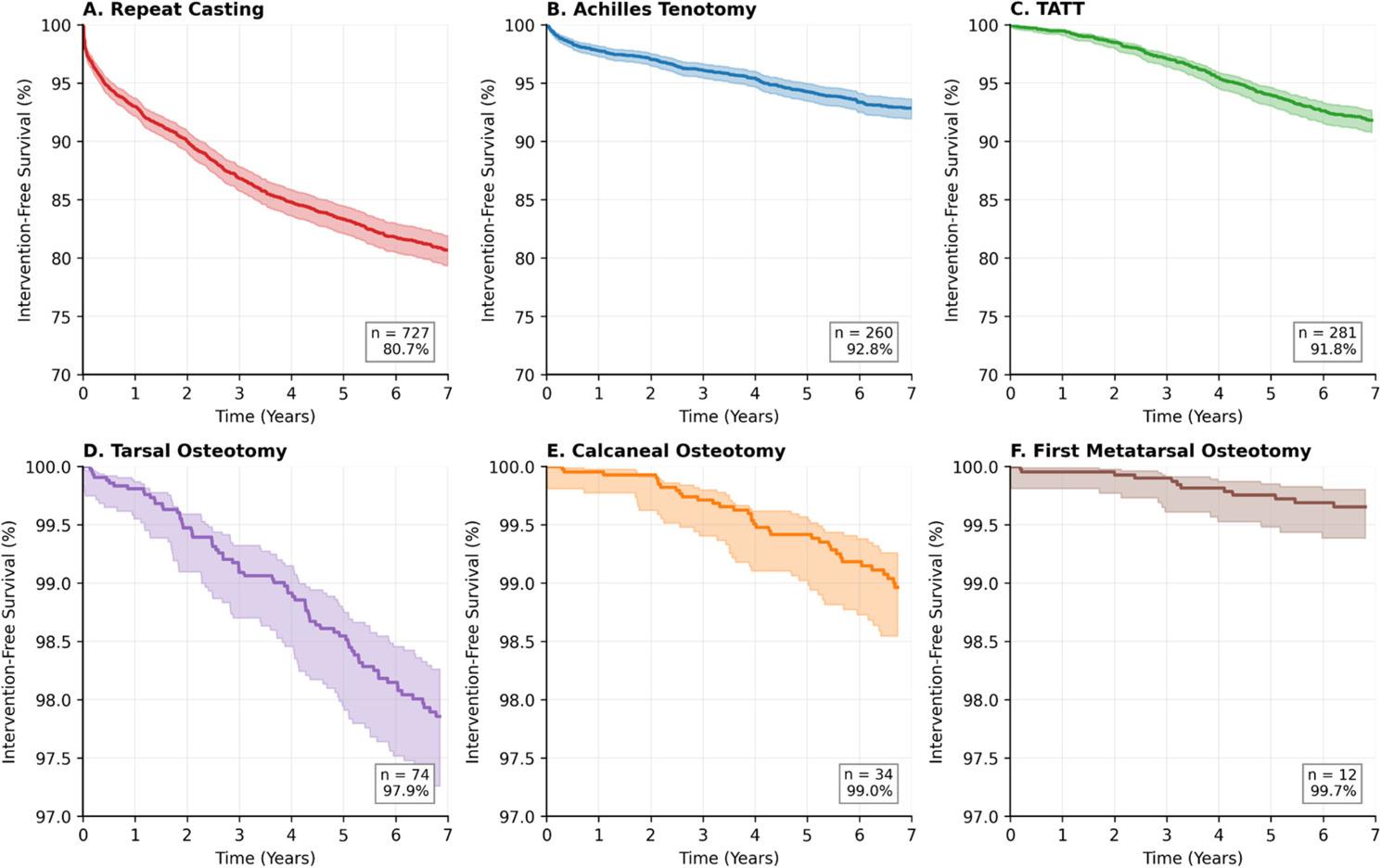
Individual Kaplan-Meier estimates by relapse intervention. Kaplan–Meier curves showing the cumulative probability of remaining free from each intervention over time, including repeat casting, repeat Achilles tenotomy, Tibialis Anterior Tendon Transfer (TATT), and osteotomies. Time is measured in years from the index visit. Patients were censored at their last recorded clinical encounter. These curves represent procedure-based proxies for recurrence rather than direct measures of recurrence

Across all procedure types, Kaplan-Meier estimates demonstrated expected temporal patterns, with casting and tendon procedures occurring earlier and more frequently, while osteotomies represented later and uncommon interventions (Fig. 3). Among children who underwent intervention, the median time to any relapse intervention was 2.3 years (IQR 0.9–3.9 years) from the index visit. Repeat casting occurred at a median of 2.1 years (IQR 0.7–3.7 years), Achilles tenotomy at 2.2 years (IQR 0.4–4.1 years), and TATT at 3.6 years (IQR 2.1–4.9 years). Bony procedures were infrequent and occurred later in the follow up period, with tarsal osteotomy performed at a median of 3.7 years (IQR 1.9–5.1 years), calcaneal osteotomy at 4.0 years (IQR 2.6–5.6 years), and first metatarsal osteotomy at 3.3 years (IQR 2.4–5.1 years). Collectively, these findings illustrate the utilization and temporal distribution of relapse management strategies following early clubfoot diagnosis and highlight the predominance of repeat conservative measures relative to operative salvage.

### Kaplan-Meier analysis

**Fig. 4:**
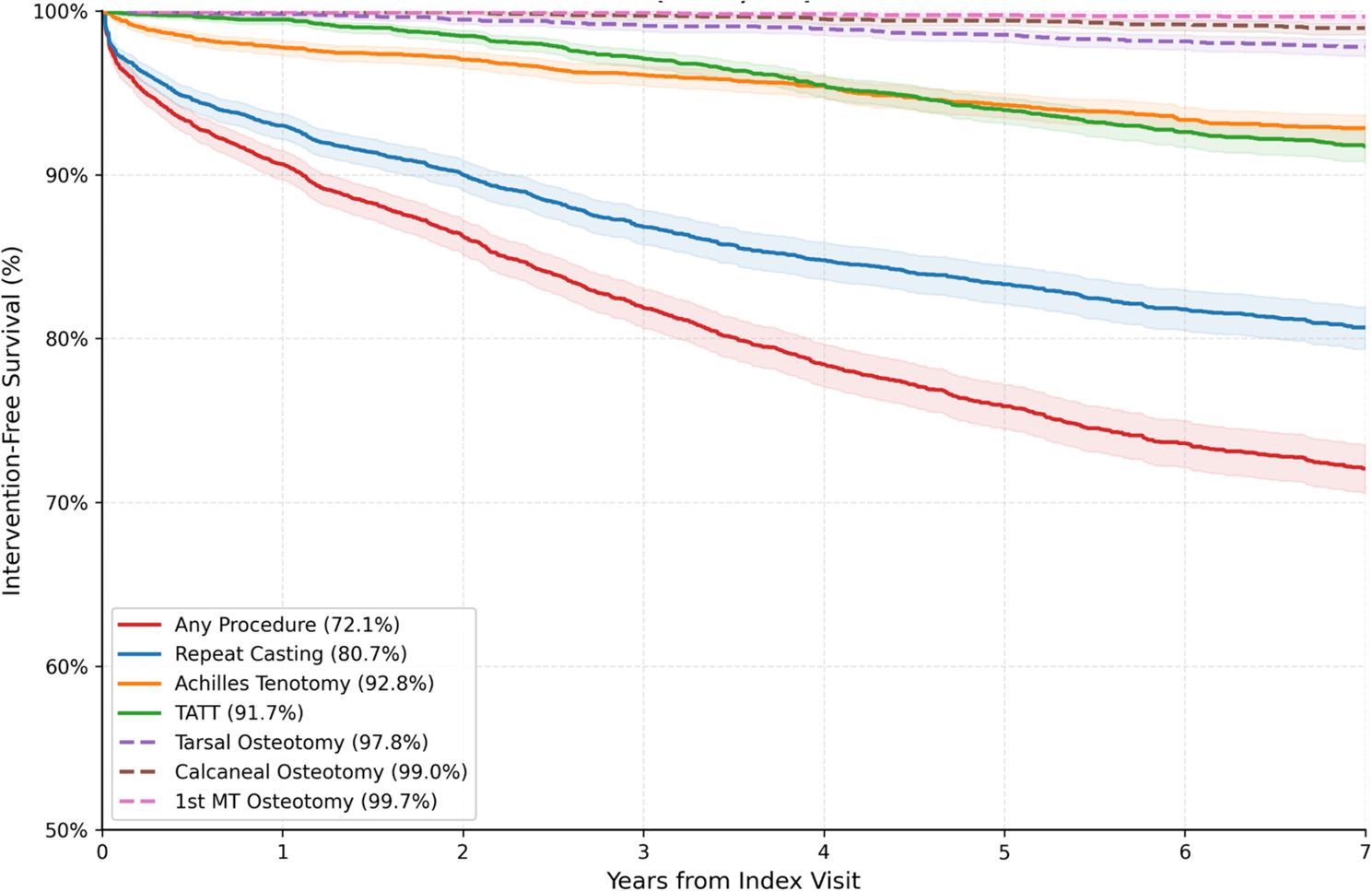
Cumulative Kaplan-Meier estimates of clubfoot relapse interventions. Kaplan–Meier curve demonstrating the cumulative probability of remaining free from any relapse-related procedure over the 7-year follow-up period. The composite outcome includes repeat casting, repeat Achilles tenotomy, Tibialis Anterior Tendon Transfer (TATT), and all osteotomy procedures. Patients were censored at their last recorded encounter. Estimates account for differential follow-up time and represent intervention-based proxies for recurrence

Kaplan-Meier survival analysis was performed to estimate the probability of remaining free from each intervention over the 7-year follow-up period, accounting for patients censored at their last clinical encounter (Fig. 4). Intervention-free survival probabilities at the end of follow-up were: 72.06% (95% CI 70.6–73.5%) for any relapse procedure, 80.68% (95% CI 79.4–81.9%) for repeat casting, 92.85% (95% CI 91.9–93.7%) for repeat Achilles tenotomy, 91.77% (95% CI 90.8–92.7%) for tibialis anterior tendon transfer, 97.82% (95% CI 97.3–98.3%) for tarsal osteotomy, 98.96% (95% CI 98.5–99.3%) for calcaneal osteotomy, and 99.65% (95% CI 99.4–99.8%) for first metatarsal osteotomy. Together, these estimates represent an approximate combined osteotomy event burden of 3.6%, supporting that most children remained free from bony salvage procedures through seven years of follow up. These Kaplan–Meier survival estimates differ from the simple proportions reported above because they account for differential follow-up time and censoring, estimating the cumulative probability of an event over time rather than the observed proportion of patients undergoing a procedure within the available follow-up period.

## Discussion

In this large multi-institutional cohort of 4,522 children with early-diagnosed clubfoot, relapse-related interventions were common. Overall, 23.11% of children underwent any relapse procedure during follow up. Repeat casting was the most common intervention, occurring in 16.08% of children, followed by TATT in 6.23% and repeat Achilles tenotomy in 5.75%. Bony procedures such as calcaneal, tarsal, and first metatarsal osteotomies were used far less frequently, representing fewer than 2% of cases. These findings provide a contemporary, population-level estimate of national practice patterns in addressing deformity requiring additional intervention.

In addition to defining the frequency of relapse-related interventions, this study provides insight into their temporal distribution. Interventions consistent with relapse occurred early in childhood, with a median time to any intervention of 2.3 years (IQR 0.9–3.9 years) from the index visit. Repeat casting and Achilles tenotomy occurred earliest, while TATT and bony osteotomies were performed later, reflecting their role in addressing more persistent or rigid deformities. This temporal pattern aligns with contemporary treatment paradigms in which early deformity requiring intervention is typically managed conservatively, reserving tendon and bony procedures for select patients with later or multiply recurrent deformity.

These findings suggest that the modern management of idiopathic clubfoot requiring repeat intervention predominantly relies on conservative management. We report rates of 16.08% which is somewhat lower than other cohorts reporting rates between 18.5 and 24.4%, potentially reflecting differences in procedural methodologies or philosophies between institutions [11, 14, 15]. Repeat tenotomy was not routinely performed with repeat casting as only 5.75% of patients within this cohort underwent this intervention. This rate is comparable if not slightly lower compared to existing literature that notes the use of repeat tenotomy between 6.6 and 14.4% of patients [14, 15]. Overall, this data reinforces the use of serial casting in the Ponseti Method’s long-term maintenance phase and its role as the most common intervention in deformity requiring additional treatment.

Interestingly, 6.23% TATT utilization in this national cohort was much lower than many single-center reports, where rates range from 12.1–29% [12, 13, 16]. This discrepancy may partly reflect coding-related undercapture in administrative data. TATT may be bundled with other reconstructive procedures, coded inconsistently across health systems, or captured under alternative procedural descriptors rather than CPT 27,691 alone. These factors could lead to underestimation of true TATT utilization compared with single-center studies, where tendon transfers are often identified through detailed chart review or operative records. Given that the average age to undergo TATT is between 4 and 7 years old, this cohort is designed to provide adequate follow up for most transfers. Consequently, higher rates reported in smaller, single-institution series may overestimate national utilization due to referral and selection bias, or alternatively reflect variability in surgeon adoption of tibialis anterior tendon transfer across practice settings. CPT 27,691 does not distinguish between tendon transfer sites (e.g., third cuneiform versus cuboid), and variation in surgical technique and philosophy across institutions is therefore not captured in this dataset.

Bony procedures, including calcaneal, tarsal, and first-metatarsal osteotomies, were rare, each occurring in fewer than 2% of children. This aligns with contemporary understanding that osteotomies are typically reserved for rigid, late-presenting, or multiply recurrent deformities often emerging beyond early childhood [17]. Kaplan-Meier survival exceeding 97% for all osteotomy categories confirms their role as salvage interventions rather than standard relapse management.

Collectively, these results suggest a stepwise pattern of relapse-related intervention in national practice. Repeat casting was the most common intervention. Repeat tenotomy and TATT were used less frequently, while bony procedures were uncommon and appeared to be reserved for a minority of children with persistent or rigid deformity. To our knowledge, this is the largest cohort study of children with idiopathic clubfoot used to evaluate relapse-related intervention patterns. The multi-institutional design improves generalizability beyond single-center experiences and provides a broader picture of contemporary practice in the United States.

This study has several limitations inherent to retrospective analyses using administrative databases. First, identification of clubfoot and relapse-related procedures relied on ICD-10 and CPT codes, which may incompletely capture clinical nuance, deformity severity, brace compliance, and laterality. Coding variation across institutions may lead to underestimation or misclassification of certain interventions, particularly for procedures that can be performed in both clinic and operative settings or combined with other corrections; this is especially relevant for TATT, which may be bundled with other procedures or captured under alternative procedural descriptors in administrative datasets. TriNetX predominantly captures data from large academic health systems and regional health networks; representativeness of rural, community-based, or Federally Qualified Health Center settings—where access to subspecialty care and adherence patterns may differ—is uncertain.

Brace non-compliance is the leading cause of clubfoot recurrence in the Ponseti literature and represents a critical unmeasured variable in this study. The database does not capture brace use or adherence, which limits the ability to account for one of the most important determinants of relapse. Elevated rates of repeat casting observed in this cohort may therefore reflect variation in brace compliance rather than differences in deformity severity or treatment strategy. As a result, interpretation of intervention rates, particularly repeat casting, should be made with caution, as these findings may partially reflect underlying differences in adherence that cannot be captured in administrative data.

Although the requirement that the index visit occur at least one year after diagnosis provides a temporal safeguard to distinguish relapse from primary treatment, some misclassification remains possible, particularly in cases of prolonged or atypical treatment courses. TriNetX does not provide granular clinical details such as clubfoot complexity in the form of Dimeglio score, type of recurrence, specific indications for each procedure, or treatment sequencing. These factors are known to influence relapse patterns and treatment decisions and cannot be accounted for in this dataset. The database does not reliably capture whether procedures were performed on unilateral or bilateral feet, preventing analysis at the limb level. As a result, intervention rates are reported at the patient level and may underestimate the true procedural burden, particularly in patients with bilateral disease, and should be interpreted with this limitation in mind. While patients were followed for up to 2,555 days (7 years) depending on index date and data availability, this captures the most common window for relapse (ages 2–7). However, some recurrences occurring in adolescence may not yet have occurred, potentially underestimating interventions such as osteotomies. Variability in age at the index visit results in heterogeneity in the age at end of follow up, and some patients may not be observed into later childhood or adolescence, potentially leading to underestimation of late recurrences. Kaplan-Meier analyses assume that loss to follow-up is non-informative; however, in this dataset, patients who transfer care to non-participating institutions are censored at their last recorded encounter, which may not be random and could lead to underestimation of intervention rates if these patients subsequently undergo relapse-related procedures elsewhere. These Kaplan–Meier survival estimates differ from the simple proportions reported above because they account for differential follow-up time and censoring, estimating the cumulative probability of an event over time rather than the observed proportion of patients undergoing a procedure within the available follow-up period. Additionally, the database does not capture whether initial treatment followed formal Ponseti protocols and recurrence itself could not be directly measured, as procedural utilization was used as a proxy (Fig. 1, 2, 3 and 4).

## Conclusion

In this large retrospective descriptive study of children with early diagnosed clubfoot, 23.11% of patients underwent additional interventions consistent with recurrence during longitudinal follow up, most commonly within 2.3 years of diagnosis. Repeat casting was the predominant management strategy, occurring in 16.08% of children, followed by repeat Achilles tenotomy in 5.75% and TATT in 6.23%. Bony procedures, including calcaneal, tarsal, and first metatarsal osteotomies, were rare, each occurring in fewer than 2% of patients and typically later in childhood. These population level estimates define the frequency and timing of contemporary intervention patterns following initial Ponseti treatment and provide clinically meaningful benchmarks for counseling families, guiding expectations, and contextualizing single institution experiences.

## Data Availability

The data analyzed during the current study were accessed through the TriNetX Research Network user interface. No line-level patient data were downloaded, exported, or accessed by the authors. The underlying patient-level data are maintained by TriNetX and participating health care organizations and are not publicly available due to data use restrictions and patient privacy protections. Aggregate results generated from the TriNetX platform are included in this published article, including the tables and figures. Researchers with appropriate institutional access to TriNetX may be able to reproduce the analyses within the platform, subject to TriNetX access requirements.

## Abbreviations

CPT: Current Procedural Terminology
HIPAA: Health Insurance Portability and Accountability Act
ICD-9: International Classification of Diseases, Ninth Revision
ICD-10: International Classification of Diseases, Tenth Revision
IRB: Institutional Review Board
STROBE: Strengthening the Reporting of Observational Studies in Epidemiology
TATT: Tibialis anterior tendon transfer
